# Correction to: Male Obesity: Epigenetic Origin and Effects in Sperm and Offspring

**DOI:** 10.1007/s40610-017-0084-4

**Published:** 2018-01-15

**Authors:** Sam Houfflyn, Christophe Matthys, Adelheid Soubry

**Affiliations:** 1 Epidemiology Research Unit, Department of Public Health and Primary Care, University of Leuven, 3000 Leuven, Belgium; 2 Clinical and Experimental Endocrinology, Department of Chronic Diseases, Metabolism, and Ageing, KU Leuven University, Leuven, Belgium

**Correction to: Curr Mol Bio Rep (2017) 3:288–296**

10.1007/s40610-017-0083-5

The article Male Obesity: Epigenetic Origin and Effects in Sperm and Offspring, written by Sam Houfflyn, Christophe Matthys, and Adelheid Soubry, was originally published Online First without open access. After publication in volume 3, issue 4, page 288–296 the author decided to opt for Open Choice and to make the article an open access publication. Therefore, the copyright of the article has been changed to © The Author(s) 2018 and the article is forthwith distributed under the terms of the Creative Commons Attribution 4.0 International License (http://creativecommons.org/licenses/by/4.0/), which permits use, duplication, adaptation, distribution and reproduction in any medium or format, as long as you give appropriate credit to the original author(s) and the source, provide a link to the Creative Commons license, and indicate if changes were made.

